# Low‐dose melatonin for sleep disturbances in early‐stage cirrhosis: A randomized, placebo‐controlled, cross‐over trial

**DOI:** 10.1002/jgh3.12356

**Published:** 2020-05-18

**Authors:** Arjuna P De Silva, Madunil A Niriella, Dileepa S Ediriweera, Jerome P De Alwis, Isurujith K Liyanage, Ushanthani Ettickan, Kasun V Liyanapathirana, Chandimani Undugodage, H. Asita de Silva, H. Janaka de Silva

**Affiliations:** ^1^ Faculty of Medicine University of Kelaniya Ragama Sri Lanka; ^2^ University Medical Unit Colombo North Teaching Hospital Ragama Sri Lanka; ^3^ Faculty of Medical Sciences University of Sri Jayawardenapura Nugegoda Sri Lanka

**Keywords:** cirrhosis, clinical trial, melatonin, sleep disturbances, treatment

## Abstract

**Background and aim:**

Melatonin is used to treat sleep disturbances (SDs). The aim of this study was to investigate the safety and efficacy of low‐dose melatonin for SDs in early‐stage cirrhosis.

**Methods:**

In a single‐center, randomized, double‐blind, placebo‐controlled, cross‐over clinical trial, patients with early‐stage (Child‐Turcotte‐Pugh [CTP] class A or B) cirrhosis with SDs, without hepatic encephalopathy, were randomized to placebo or 3 mg of melatonin for 2 weeks. After 2 weeks, the patients were given a washout period of 1 week and crossed over to melatonin or placebo for a further 2 weeks. The Pittsburgh Sleep Quality Index (PSQI) and Epworth Sleepiness Scale (ESS) were used to measure sleep quality and daytime sleepiness, respectively. Analysis of results was based on intention to treat, and linear mixed‐effect models were used to evaluate the effect of melatonin. Analysis was conducted using R‐programming language 3.5.1.

**Results:**

Seventy one patients were recruited (mean age: 61.9 ± 8.7 years, males: 46 [64.8%], and CTP Class A = 52 [73.2%] and Class B = 19 [26.8%]). Sixty patients completed the study (mean age: 61.7 ± 8.8 years, males: 40 [66.6%], and CTP Class A = 45 [75.0%] and Class‐B = 15 [25.0%]). Two patients dropped out due to adverse events. Nine patients were lost to follow up. Patients given melatonin had a significantly lower PSQI and ESS compared to both pretreatment (*P* < 0.001) and postplacebo scores (*P* < 0.001). Incidence of adverse events was similar (two each of abdominal pain, one each of headache, one each of dizziness) in both groups.

**Conclusion:**

Melatonin seems safe and effective for use in patients with SDs in early‐stage cirrhosis in the short term. However, larger and longer‐term studies to assess efficacy and safety are required before its clinical use can be recommended.

## Graphical abstract

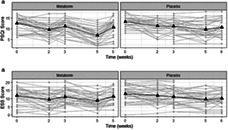

This is a single‐centre, randomized, double‐blind, placebo‐controlled, cross‐over clinical trial, on patients with early‐stage [Child‐Turcotte‐Pugh class A or B] cirrhosis with sleep disturbances and without hepatic encephalopathy. The aim of this study was to assess the safety and efficacy of administrating 3mg of melatonin for two weeks on improving sleep disturbances. Melatonin seems safe and effective for use in patients with sleep disturbances in early‐stage cirrhosis, in the short term.

## Introduction

Sleep disturbance (SD), unsatisfactory sleep, and altered sleep patterns are common among patients with cirrhosis. Up to 40–50% of patients with cirrhosis report poor and unsatisfactory sleep.^1^, ^2^, ^3^ Reduced sleep is associated with psychological problems, which may result in anxiety and depression^4^ and a poor quality of life. It can also have wide‐ranging effects on the cardiovascular, endocrine, immune, and nervous systems, with a significant socioeconomic impact.^5^, ^6^

Prolonged sleep latency, reduced duration and quality of sleep, daytime somnolence, and frequent awakening during sleep are common sleep disorders in patients with cirrhosis.^7^ A case–control study using polysomnographic assessment of patients with cirrhosis and healthy volunteers showed reduced sleep efficiency in 73% of patients with cirrhosis; the mean time to fall asleep (sleep latency) was 151 min in patients with cirrhosis compared with 90 min in healthy adults. Impairment of the sleep–wake cycle is one of the early manifestations of hepatic encephalopathy (HE)^8^, ^9^ leading to night‐time insomnia and daytime drowsiness.^1^, ^10^ These disorders are significantly more common in patients with advanced (Child‐Turcotte‐Pugh [CTP] C) cirrhosis compared to patients with CTP A and B cirrhosis.^11^

In patients with cirrhosis, hypnotic agents such as benzodiazepines and zolpidem, which act on the GABAergic neurons, may cause HE and are therefore of limited use. In a randomized, double‐blind trial on 35 patients with cirrhosis, hydroxyzine, an H1 receptor antagonist, demonstrated improvement in quality of sleep.^12^ In another trial in patients with minimal HE, lactulose was found to improve sleep parameters.^13^ Nevertheless, there is no clear evidence of the effectiveness and safety of these pharmacological agents in the management of SD in cirrhosis.^4^, ^14^, ^15^

The exact mechanism of SD in patients with cirrhosis is poorly understood. Patients with cirrhosis are known to have an altered circadian rhythm of serum melatonin secretion, where the peak plasma levels are achieved later than in normal healthy adults.^16^ In addition, hepatic metabolism of melatonin is decreased in patients with cirrhosis, and the basal level of melatonin is higher, and suppression of melatonin to light is blunted. These alterations may contribute to SD in cirrhosis.^16^, ^17^

We, therefore, investigated the safety and efficacy of melatonin for SD among patients with early‐stage cirrhosis (CTP A and B).

## Methods

### *Study setting and patient population*

We conducted a single‐center, randomized, double‐blind, placebo‐controlled, cross‐over clinical trial to assess the efficacy and safety of low‐dose melatonin in improving SD among patients with early‐stage (CTP class A or B) cirrhosis. This study was carried out in the gastroenterology outpatient clinic of the University Medical Unit, Colombo North Teaching Hospital, Ragama, Sri Lanka, between October 2018 and April 2019.

We recruited consecutive, consenting patients, aged 18–65 years, with CTP class A or B cirrhosis who complained of SD. The diagnosis of cirrhosis was based on compatible clinical, biochemical, and radiological features. Those with present or prior overt HE, respiratory or neurological disorders, or other diseases or treatments that could affect sleep were excluded. Patients with obstructive sleep apnea, diagnosed by either neck circumference >17 inches for men and >16 inches for women or a positive Berlin questionnaire, and those having evidence of moderate to severe depression on the Beck Depression Inventory (BDI) (score ≥20) were also excluded.

### *Study design*

**Figure 1:**
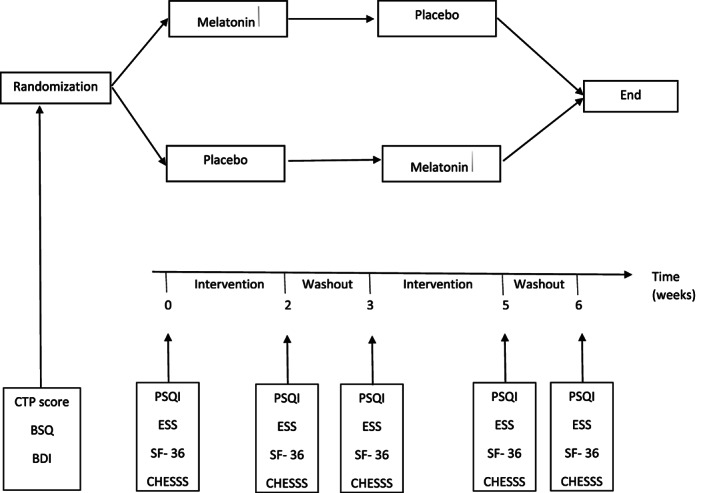
Study protocol.

A 3 mg melatonin tablet, administered orally as a single dose at the same time every day before bed, was used as the active agent. A matching placebo tablet containing starch was used as the control. Selected patients were either randomized to receive melatonin (active phase) or placebo (control phase) daily for 2 weeks. After 2 weeks, the patients were given a washout period of 1 week and then crossed over to drug or placebo for another 2 weeks (Fig. 1).

Randomization was carried out centrally, where each patient was assigned an unmarked study pack in a sequential manner. Each study pack contained two similar pill bottles marked with serial numbers, containing the active drug and the placebo. The contents of the pill bottles were not identifiable to the patient, treating clinician, or the investigator. Patients were randomly given a bottle for the first phase and the remaining bottle for the second phase. The contents in the bottle were only known when the serial numbers were decoded at the completion of the study.

### *Study outcomes*

The primary outcome measure was improvement in sleep quality measured by the Pittsburgh Sleep Quality Index (PSQI), and the secondary outcomes measured were reduction in daytime sleepiness measured by the Epworth Sleepiness Scale (ESS), quality‐adjusted life years (QALY) measured by the Short Form Health Survey (SF 36), and the presence of HE measured by the clinical hepatic encephalopathy scoring system (CHESS). To detect a 20% improvement in PSQI with a two‐sided confidence interval of 95% and 80% power for equal exposed and unexposed samples, the sample size was calculated to be 118 (59 participants to complete initial and cross‐over phases).^18^ Participants were asked to maintain a sleep diary on a daily basis and were assessed at baseline, 2 weeks after intervention, 1 week after washout, and 2 weeks after cross‐over in the outpatient clinic. PQSI and ESS were used to measure sleep quality and daytime sleepiness, respectively, at baseline, at end of intervention and washout for both melatonin and placebo, and at completion of the study (Fig. 1).

### *Statistical analysis*

Baseline characteristics of the patients who initially received melatonin and placebo were compared with a two‐sample *t*‐test, assuming equal variances as appropriate, for continuous variables and Pearson's chi‐square test and Fisher's exact test, as appropriate, for categorical variables. We evaluated the pre and post measurements of the ESS, PSQI, and SF 36 components as outcomes between melatonin and placebo using a paired‐sample *t*‐test. The outcomes that showed significant differences in pre‐ and postinterventional measurements were then considered for subsequent analysis to evaluate the effect of melatonin.

Outcomes of each patient had been measured five times during this study (i.e. before the intervention, after the intervention of first cycle, after the washout period of the first cycle, after the intervention of the cross‐over cycle, and after the washout period of the cross‐over cycle). Therefore, we considered time an explanatory variable in the analysis as the outcomes were longitudinally measured in each individual. These outcomes were measured during the periods of intervention (i.e. melatonin or placebo) and nonintervention (i.e. baseline and washout); therefore, we defined our intervention as a categorical variable with three levels (i.e. melatonin, placebo, and no intervention). Initially, we developed separate linear models for ESS and PSQI measurements considering time and intervention as independent variables. Subsequently, we investigated for unexplained variability of the fitted linear models by fitting linear mixed‐effects models by incorporating individual‐level random intercepts. Mixed‐effect models were fitted with lme4 package by maximizing log‐likelihood. In the model, we included lactulose and rifaximin as potential confounders for ESS and PSQI scores. Inclusion of individual‐level random effects and confounders in the final models was determined by evaluating the log‐likelihood ratio test. Analysis of results was based on intention to treat (ITT), where missing values of the patients who dropped out and were lost to follow up were replaced with the most recent available follow‐up values. Analysis was conducted using R programming language 3.5.1. A *P* value <0.05 was considered statistically significant.

### *Ethical considerations*

Ethics approval for the trial was obtained from the Ethics Review Committee of the Faculty of Medicine, University of Kelaniya, Ragama, Sri Lanka (P/152/08/2018). The trial was registered in the Clinical Trials Registry of Sri Lanka (APPL/2019/006).

## Results

### *Patient demographics*

**Figure 2:**
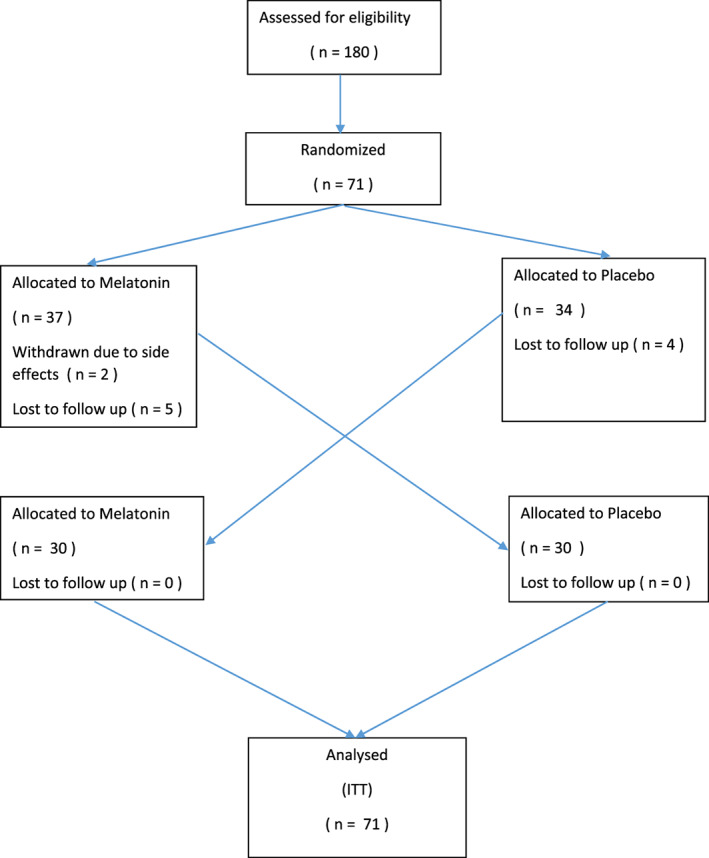
CONSORT diagram.

Seventy‐one patients were recruited to the study (mean age 61.9 ± 8.7 years, males: 46 [64.8%], and CTP Class A = 52 [73.2%] and Class B = 19 [26.8%]). The baseline characteristics of the patients who are initially allocated to melatonin and placebo groups are presented in Table 1. Two patients dropped out due to melatonin adverse events, and nine patients were lost to follow up. Therefore, 30 patients each were allocated to each arm, completing the prespecified 59 interventions required to power the primary outcome of the study (Fig. 2). Sixty patients completed the study (mean age 61.7 ± 8.8 years, males: 40 [66.6%], and CTP Class A = 45 [75.0%] and Class B = 15 [25.0%]).

**Table 1:** Baseline characteristics of the study population at inclusion

	Melatonin (*n* = 37) mean (SD) or number (%)	Placebo (*n* = 34) mean (SD) or number (%)
Age	63.6 (9.7)	60.1 (7.1)
Male gender	21 (56.7%)	25 (73.5%)
Married	36 (97.3%)	32 (94.1%)
Educational level above secondary	24 (64.9%)	19 (55.9%)
Monthly income (Rs)	27 000 (13 900)	31 600 (23 000)
Beck depression inventory (BDI)	11.8 (4.5)	10.7 (4.3)
Weight (kg)	61.6 (8.9)	61.0 (9.8)
Child‐Turcotte‐Pugh (CTP) class A	26 (70.3%)	26 (76.5)
Model for end‐stage liver disease (MELD)	11.6 (3.5)	11.1 (3.2)
Presence of varices	26 (70.3%)	21 (61.8%)
Lactulose use	32 (91.4%)	20 (58.8%)
Rifaximin use	4 (11.4%)	5 (14.7%)

The incidence of self‐reported adverse events were minor and were similar (two patients each for abdominal pain, *P* = 1.00; one patient each for headache, *P* = 1.00; and one patient each for dizziness, *P* = 1.00) during melatonin and placebo administrations (Table 2). None of the participants had precipitation of HE with 2 weeks' treatment of melatonin.

**Table 2:** Adverse events and discontinuation of drug and placebo among the participants

	Melatonin (*n* = 37) number (%)	Placebo (*n* = 34) number (%)	*P* value
Abdominal pain	1 (2.7%)	1 (2.9%)	1.00
Headache	1 (2.7%)	0 (0.0%)	1.00
Dizziness	1 (2.7%)	0 (0.0%)	1.00
Discontinuation	7 (18.9%)	4 (11.8%)	0.518

### *Outcome analysis*

**Figure 3:**
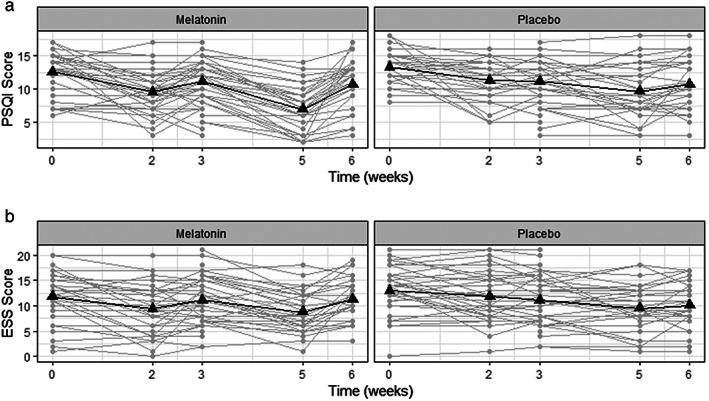
(a) Changes in Pittsburgh Sleep Quality Index (PSQI) and (b) Epworth Sleepiness Scale (ESS) of melatonin and placebo groups during the study period. Gray points interconnected by lines indicate individual patient responses during the follow up, and black triangles interconnected by lines indicate the mean response in the respective groups.

Pre‐ and postadministration measurements of PSQI score, ESS score, and SF 36 questionnaire for the melatonin and placebo groups are shown in Table 3. Both the melatonin and placebo groups showed significant differences in pre‐ and postadministration measurements of ESS and PSQI scores. None of the SF 36 components showed differences in pre‐ and postadministration melatonin and placebo measurements, except for SF 1 in the placebo group. Therefore, we considered only PSQI and ESS scores for further analysis. The changes in PSQI and ESS scores during melatonin and placebo administration are shown in Figure 3. Patients given melatonin showed a clear decrease in post‐treatment PSQI and post ESS scores compared with placebo with respect to their pretreatment and washout values.

**Table 3:** Preadministration and postadministration scores of Pittsburgh Sleep Quality Index (PSQI) and Epworth sleepiness scale (ESS) and Short Form Health Survey (SF 36) among melatonin and placebo groups

	Melatonin mean (SD)			Placebo mean (SD)		
Indicator	Preadministration score	Postadministration score	*P* value	Preadministration score	Postadministration score	*P* value
PSQI score	12.6 (3.1)	9.6 (3.1)	<0.01	11.4 (2.9)	7 (3.4)	<0.01
ESS score	11.8 (4.7)	9.4 (4.9)	<0.01	11.7 (4.5)	8.7 (4.0)	0.01
SF1	59.1 (24.3)	57.2 (26)	0.45	65.7 (20.6)	68.8 (20.0)	<0.01
SF2	14.9 (24.6)	17.6 (31.1)	0.50	19.1 (32.6)	28.3 (37)	0.08
SF3	27.9 (41.2)	32.4 (42.7)	0.28	43.1 (41.5)	53.3 (41.6)	0.16
SF4	49.3 (20.0)	53 (17.1)	0.11	61.8 (14.3)	63.7 (14.9)	0.08
SF5	57.6 (14.9)	59.8 (13.9)	0.21	65.3 (13.9)	67.9 (14.3)	0.14
SF6	60.1 (22)	61.4 (21.7)	0.47	65.4 (18.0)	67.9 (16.3)	0.12
SF7	50.6 (28.5)	52.6 (24.4)	0.36	57.5 (22.2)	59.6 (21.7)	0.54
SF8	32.2 (16.9)	35.5 (14.7)	0.05	42.5 (14.0)	43.5 (13.8)	0.89
SF9	49.3 (29.7)	48 (26.6)	0.42	57.1 (26.3)	57.5 (27.2)	0.92

### *Pittsburgh Sleep Quality Index*

The fixed‐effect linear model showed that time and intervention are significantly associated with PSQI scores of the patients. The variance of the individual‐level random effect was significantly different from zero (Δ deviance 212.8, degree of freedom [*df*] 1, *P* < 0.001), and a mixed‐effect linear model was chosen. Lactulose showed a significant reduction in the log likelihood of the fitted mixed‐effect linear model and was included (Δ deviance 35.0, *df* 1, *P* < 0.001) (Table 4).

**Table 4:** Parameter estimates of the fitted mixed‐effect model for Pittsburgh Sleep Quality Index (PSQI) score and Epworth Sleepiness Scale (ESS) scores

	Estimate	Std. Error	*t* value	*P* value
PSQI score				
Intercept	12.36	0.67	18.50	<0.001
Time (weeks)	−0.44	0.05	−7.96	<0.001
Intervention				
No intervention *vs* melatonin	−2.99	0.29	−10.40	<0.001
No intervention *vs* placebo	−0.81	0.29	−2.79	0.005
Placebo *vs* melatonin	−2.18	0.36	5.96	<0.001
Lactulose	0.60	0.74	0.80	0.424
Variance (random effects)	6.33			
Variance (residuals)	4.25			
ESS score				
Intercept	13.67	0.99	13.76	<0.001
Time (weeks)	−0.34	0.06	−5.70	<0.001
Intervention				
No intervention *vs* melatonin	−2.16	0.31	−6.99	<0.001
No intervention *vs* placebo	−0.39	0.31	−1.27	0.205
Placebo *vs* melatonin	−1.76	0.39	4.48	<0.001
Lactulose	−1.88	1.12	−1.67	0.099
Variance (random effects)	15.32			
Variance (residuals)	4.90			

According to the model, the PSQI score showed an average reduction of 0.44 (95% confidence interval [CI]: 0.32–0.54) per week during the follow‐up period irrespective of the intervention (*P* < 0.001). Melatonin reduced the PSQI score by an average of 2.99 (95% CI: 2.42–3.55), and this reduction was significant compared to the preintervention stage (*P* < 0.001). There was a reduction of PSQI score by an average of 0.81 (95% CI: 0.24–1.37) in the placebo group, which was also lower than the preintervention stage (*P* = 0.005). The reduction of PSQI score in the melatonin group was significantly higher than the reduction observed in the placebo group (i.e. average difference 2.18; 95% CI: 1.46–2.90; *P* < 0.001).

### *Epworth Sleepiness Scale*

The fixed‐effect linear model showed that the time and intervention were significantly associated with ESS scores of the patients. The variance of the individual‐level random effects showed significant difference from zero (Δ deviance 212.8, *df* 1, *P* < 0.001), and a mixed‐effect linear model was adopted. Lactulose showed a significant reduction in the log likelihood of the fitted mixed‐effect linear model and was included (Δ deviance 35.0, *df* 1, *P* < 0.001) (Table 4).

According to the model, the ESS score showed an average reduction of 0.34 (95% CI: 0.22–0.45) per week during the follow‐up period irrespective of the intervention (*P* < 0.001). Melatonin reduced the ESS score by an average of 2.16 (95% CI: 1.55–2.77), and this reduction in score was significant compared to the preintervention stage (*P* < 0.001). Placebo reduced the score only by 0.39 (95% CI: −0.22 to 1.01), which was not different from the preintervention stage (*P* = 0.205). Melatonin reduced ESS score by an average of 1.76 (95% CI: 0.99–2.53) compared to placebo (*P* < 0.001).

## Discussion

Patients given melatonin had significantly lower PSQI and ESS scores compared to both pretreatment and post‐placebo scores. Therefore, melatonin improved sleep quality and reduced daytime sleepiness in patients with early‐stage cirrhosis. Melatonin was also well tolerated, and there were very few reported adverse events. Melatonin use in the short term seems safe and effective as a sedative to treat SD in patients with CTP classes A and B cirrhosis. To our knowledge, this is the first randomized, double‐blinded, placebo‐controlled clinical trial (RCT) to assess the efficacy and safety of melatonin in treating SD in early‐stage cirrhosis. The trial was not sponsored by industry.

The normal circadian rhythm of the secretion of melatonin is altered in cirrhotic patients, resulting in an inverse pattern, with high daytime levels.^16^, ^17^ As the hepatic metabolism of melatonin is decreased in patients with cirrhosis, this elevation is related to the degree of liver failure.^19^ Although SD in cirrhosis cannot be attributed to only the alteration in the circadian rhythm of melatonin and is probably multifactorial,^20^ deranged melatonin metabolism is likely to contribute significantly to SD in cirrhosis.^16^, ^17^

We adopted linear mixed‐effect models in this study. The individual‐level random intercepts accommodate individual‐level variations in PSQI and ESS responses during the follow‐up period. We included time as an independent variable to detect any linear trend in PQSI and ESS values during the follow‐up period. Both PSQI and ESS scores showed reduction of scores during the follow‐up period in the entire cohort irrespective of the intervention. After adjusting for individual‐level variations and the reduction of scores over the follow‐up time, only melatonin showed a significant reduction in both PSQI and ESS scores compared to preintervention and postplacebo values. The placebo group showed reduction in PSQI scores compared to preintervention; however, the melatonin showed a higher reduction compared to placebo. There was no difference in ESS among the placebo group. Group comparison stage showed a reduction in both postintervention PSQI and ESS values compared to preintervention PSQI and ESS values in both the melatonin and placebo groups. The reduction in ESS in the placebo group can be attributed to the reduction in ESS scores during the follow‐up period rather than to an interventional effect.

In this study, PQSI and ESS were used to measure sleep quality and daytime sleepiness, respectively. The PSQI is a tool that is commonly used in clinical trials to assess the quality of sleep.^21^, ^22^, ^23^, ^24^ It calculates a score ranging from 0 to 21, and values of more than 5 indicate poor quality of sleep, with a diagnostic sensitivity of 89.6% and specificity of 86.5%. PSQI has been validated in patients with different conditions, including malignancy, chronic obstructive airway disease, and pregnancy, and in the elderly. The two scores have also been used to assess sleep in patients with cirrhosis.^25^ Although the original version of the PSQI was designed to measure sleep reports over a 1‐month period, the PSQI has been used for shorter time intervals.^21^, ^23^, ^26^ The ESS is a simple questionnaire that measures the general level of daytime sleepiness.^27^, ^28^ ESS scores significantly correlate with sleep latency measured by the multiple sleep latency test and during overnight polysomnography. Quality‐of‐life assessment was a secondary outcome measurement in our study. We could not observe differences in the quality of life except for the SF 1 component in the placebo group. This can probably be explained by the relatively short duration of our study.

The use of melatonin and its receptor agonists are well established for primary and secondary sleep disorders such as jetlag and insomnia in elderly patients.^28^, ^29^, ^30^ Melatonin has also been shown to improve sleep and quality of life in patients with asthma, Alzheimer's disease, and patients receiving chemotherapy.^31^, ^32^, ^33^ However, it has not been previously evaluated for the treatment of SD in cirrhosis. Sedative and hypnotic agents, such as benzodiazepines, and GABA agonists, such as zolpidem, should be avoided in the treatment of SD in cirrhosis. This is especially true in CTP class B, where use of these agents may precipitate HE.^25^ The present study provides evidence for a safe and effective alternative in early‐stage cirrhosis, although we are unable to comment on the use of melatonin in CTP class C cirrhosis or patients with a past history of overt HE as these groups of patients were excluded from the study. At recruitment, several of our patients were on lactulose for treatment of constipation, and a few had been given rifaximine even though they did not have a history of overt HE. In our statistical model, we included lactulose and rifaximin use as potential confounders of ESS and PSQI scores.

The strength of the present study was its design, with the majority of participants completing the trial protocol. There were, however, several limitations. We were unable to ascertain the reason for dropping out in patients who did not complete the study, although it was most likely due to logistical reasons where participants were unable to comply with the follow‐up protocol. We tried to negate this by using an ITT analysis. Although we excluded HE, we did not formally assess for and exclude minimal HE in this trial. We were also unable to assess sleep structure using polysomnography or actigraphy, nor assess levels of cortisol, neurohormones, and melatonin due to nonavailability and financial constraints. Furthermore, the effects of melatonin were assessed only in the short term.

In conclusion, melatonin seems safe and effective as a sedative in patient with CTP classes A and B cirrhosis and SD in the short term. This finding may have clinical applications in the holistic management of patients with cirrhosis. However, larger studies to assess the efficacy and safety of melatonin in the long term and in the later stages of cirrhosis are required before its clinical use can be recommended.
